# When Your Case Is Weak Abuse the Other Side

**Published:** 1904-05

**Authors:** 


### WHEN YOUR CASE IS WEAK ABUSE THE OTHER SIDE.

This maxim has been a favorite stand-by with the legal profession from time immemorial and unfortunately certain pharmaceutical manufacturers have recently seen fit to make use of that maxim. This is particularly true of the manufacturers of a certain iron preparation.

The impudence and effrontery with which these people try to hoodwink the medical profession is rather remarkable.

No other preparation ever came before the medical practitioner with so little detail as to methods of preparation, composition, therapeutic effect, etc., and nevertheless the profession is asked to accept the wildest and most extravagant statements as to its wonder-working capabilities. This is not all. The makers of this preparation, in seeking the support of the profession, covertly attack and sling mud at all other iron preparations that have been before the profession for years. They single out Pepto-Mangan, a combination which has stood the tests of the leaders in the scientific medical world both here and abroad, an organic iron combination in which, in its results, the general practitioner and the hospital clinician have learned from experience to place implicit confidence.

This unbusinesslike method of attempting to cast discredit upon other reliable and thoroughly tested combinations we can not term otherwise than despicable, and furthermore we know our readers can not be influenced by unsupported statements of financially interested parties, but will always bear in mind that Gude's Pepto-Mangan was submitted to the profession as an organic iron product, and the results obtained by its use, as also the scrutiny of analysis by chemists of repute, substantiate all that has ever been claimed for it.

Attempting to foist upon the attention of the physician a product simply by insinuation that known articles are inferior is a manner of doing business which should receive the stamp of disapproval by every one of our profession.—*Editorial in the Toledo Medical and Surgical Reporter, April, 1904.*

